# Racial/Ethnic Disparities Among US Children and Adolescents in Use of Dental Care

**DOI:** 10.5888/pcd17.190352

**Published:** 2020-07-30

**Authors:** Valerie Robison, Liang Wei, Jason Hsia

**Affiliations:** 1 Division of Oral Health, Centers for Disease Control and Prevention, Atlanta, Georgia; 2 DB Consulting Group, Inc, Atlanta, Georgia; 3 Division of Population Health, Centers for Disease Control and Prevention, Atlanta, Georgia

## Abstract

**Introduction:**

Dental care among children has increased over the past decade, and racial/ethnic disparities have narrowed for some groups. We measured changes in racial/ethnic disparities in annual dental care for children and adolescents aged 2 to 17 years and conducted multivariate analysis to study factors associated with changes in disparities over time.

**Methods:**

We used Medical Expenditure Panel Survey data to obtain crude prevalence estimates of dental care use and calculated absolute disparities and changes in disparities for 3 racial/ethnic groups of children and adolescents compared with non-Hispanic white children and adolescents relative to fixed points in time (2001 and 2016). We pooled all single years of data into 3 data cycles (2001–2005, 2006–2010, and 2011–2016) and used multivariate regression to assess the relationship between dental care use and race/ethnicity, controlling for the covariates of age, sex, parents’ education, household income, insurance status, and data cycle (time).

**Results:**

Use increased by 18% only in low-income children and adolescents. Low-income Hispanic (adjusted prevalence ratio [aPR] = 0.98; 95% CI, 0.94−1.02) and Asian (aPR = 0.92; 95% CI, 0.83−1.02) participants showed no difference in dental care use relative to non-Hispanic white participants, but non-Hispanic black participants had significantly lower use (aPR = 0.84; 95% CI, 0.81−0.88). Public and private insurance were associated with a doubling of use among low-income children.

**Conclusion:**

We saw a modest increase in dental care use and a narrowing of disparities for some low-income children and adolescents. Use among low-income Hispanic and Asian participants “caught up” with use among Hispanic white participants but remained well below that of children and adolescents in families with middle and high incomes. Disparities persisted for non-Hispanic black participants at all income levels.

**Summary**

**What is already known about this topic?**

Dental care among children has increased over the past decade, and racial/ethnic disparities have narrowed for some groups.

**What is added by this report?**

We used crude prevalence estimates of dental care use to calculate absolute disparities and changes in disparities. We used multivariate analysis to determine factors associated with changes in disparities from 2001 through 2016. We included Asians, for whom many disparity studies have not had sufficient data.

**What are the implications for public health practice?**

Our study adds to the few long-term, controlled studies of dental care use by using a national data set representative of US children and adolescents.

## Introduction

Racial and ethnic disparities in children’s oral health and their access to dental care have been well documented (1–3). Findings from many studies show the highest use among non-Hispanic white populations and the lowest use among Hispanic and non-Hispanic black populations. Studies have shown an increase in use and some narrowing of racial/ethnic disparities among children and adolescents aged 2 to 17 years, but disparities relative to non-Hispanic white children persist by family income and insurance status (4–7). Studies of national survey data showed that children aged 2 to 17 years had a steady growth in annual dental visits from 1997 through 2010 (7–9). This growth is primarily due to an increase in insured children and a shift from private to public insurance as public insurance programs expanded (10,11). Public health insurance programs covering dental care expanded for low-income children through Medicaid in the 1980s and 1990s and through the enactment of the Children’s Health Insurance Program (CHIP) in the 1990s. Publicly funded dental care also expanded through the enactment and reauthorization of the State Children’s Health Insurance Program (SCHIP) in 1997 and in 2007 (12–14).

Healthy People 2020, the national framework of more than 1,200 objectives for tracking the health of Americans, focuses on achieving health equity and eliminating disparities. Each objective has a nationally representative data source, a baseline value, and a target to be reached by 2020 (15). One objective of Healthy People 2020 is to increase the proportion of children who make an annual dental visit to 49% by 2020 (16). Changes in disparities can be monitored by using methodology promoted by the Healthy People 2020 program (17–19).

We hypothesized on the basis of past studies that either non-Hispanic black or Hispanic children would have the lowest levels of changes in disparities relative to non-Hispanic white children (4–7). Our first aim was to quantify changes in disparities by using Healthy People 2020 methodology. Our second aim was to determine factors associated with changes in disparities by using multivariate analysis.

## Methods

### Data source

We analyzed data on 132,763 children by using a subset of 2001–2016 data from the Medical Expenditure Panel Survey–Household Component (MEPS-HC), a nationally representative survey managed by the Agency for Healthcare Research and Quality. MEPS-HC contains information on demographic characteristics, health status, access to care, health insurance coverage, household income, employment status of the head of household, and use of health services. Since 2001, approximately 7,300 to 9,700 children from 13,000 American families have been included in each year of MEPS. This number represents 62.7 to 64.4 million children aged 2 to 17 years in the United States (20). MEPS is conducted by using 5 rounds of in-person interviews with a study participant aged 2½ years or older. MEPS-HC is generally the data source of choice for estimating dental care use and assessing disparities, including assessments in analyses for Healthy People 2020.

### Variable definitions

The outcome variable, dental care use, was defined as prevalence as reported by a parent or caregiver of a dental visit or visits at any round of interview during the calendar year assessed with the question, “Since [START DATE]/Between [START DATE] and [END DATE], did [PERSON] see or talk to any type of dental care provider, such as the types listed on this card, for dental care or a dental check–up?” (20).

Race/ethnicities we studied were non-Hispanic white, non-Hispanic black, Hispanic, and Asian, which included Native Hawaiian and other Pacific Islanders. Other covariates were age group (2–4 y, 5–11 y, 12–17 y), sex, parent or guardian’s education (<12th grade, 12th grade, >12th grade), annual household income by percentage above the federal poverty level (FPL) (defined as low, <200%; middle, 200−399%; high, ≥400%), medical health insurance status (private, public, uninsured), dental insurance (yes/no), and data cycle (time). Medicaid, CHIP, and SCHIP were included under the public insurance category (20). American Indian and Alaska Native children were excluded from the study because of the small sample size.

### Disparities analysis

We defined a disparity as a difference in prevalence of dental care use among a racial/ethnic group relative to non-Hispanic white children. Non-Hispanic white children were selected as the reference group because they have the highest prevalence of dental care use or “most favorable” outcome (17). Disparities were measured by using single-year crude prevalence estimates for 2001 and 2016. An absolute disparity was the arithmetic difference between one group’s prevalence and the prevalence among non-Hispanic white children. A measure of change in disparities over time was the percentage-point difference between the absolute disparity at baseline (2001) subtracted from the absolute disparity at the most recent data point (2016) (18). We based tests for significant differences in use between a racial/ethnic group relative to non-Hispanic white children on a 2-sided *z* test at *P* < .05 level of significance; 95% CIs were calculated for absolute disparities. The unit of measurement for absolute disparity and change in disparity was percentage-point difference.

### Bivariate analysis

Data were pooled into 3 data cycles (2001–2005, 2006–2010, and 2011–2016), and cycles were analyzed separately. We described prevalence of population characteristics by race/ethnicity and by dental care use. We based tests of significant differences on a 2-sided *t* test at *P* < .05 level of significance, and we calculated 95% CIs.

### Multivariate analysis

We used logistic regression to study the relationship between the outcome of dental care use and race/ethnicity, controlling for covariates of age, sex, parents’ education, household income, insurance status, and data cycle (time). Adjusted prevalence ratios (aPRs) were calculated to quantify changes in use over 3 periods, controlling for other covariates. All analyses took the complex survey design into consideration by using SAS callable SUDAAN 11.0 (RTI International).

## Results

**Disparities analysis.** We found significant differences in our sample of 132,763 children in the prevalence of use among non-Hispanic black children (31.4%), Hispanic children (33.3%), and Asian children (38.1%) compared with non-Hispanic white children (56.8%) (*P* < .001) (Table 1). By 2016, dental care use increased for all groups; however, differences relative to non-Hispanic white children remained significant (*P* < .001). In 2016, all racial/ethnic groups except non-Hispanic black children (44.1%) had reached or surpassed the Healthy People 2020 target of 49%. In 2001, absolute disparities, relative to non-Hispanic white children, were 25.4 percentage points (95% CI, 20.9−29.9) for non-Hispanic black children, 23.6 percentage points (95% CI, 19.9−27.2) for Hispanic children, and 18.8 percentage points (95% CI, 11.8−25.6) for Asian children. In 2016, absolute disparities narrowed significantly for non-Hispanic black children (15.7 percentage points; 95% CI,10.5−20.9), for Hispanic children (9.1 percentage points; 95% CI, 4.0−14.1), and for Asian children (4.7 percentage points; 95% CI, 3.1−12.4). From 2001 through 2016, the measure of change in disparities relative to non-Hispanic white children showed significant decreases for non-Hispanic black children (−9.7 percentage points, *P* = .006), Hispanic children (−14.4 percentage points, *P* < .001), and Asian children (−14.1 percentage points, *P* = .008). Non-Hispanic black children had the lowest change in disparities.

**Table 1:** Crude Prevalence of Dental Care Use, Absolute Disparity, and Change in Disparity Among US Children and Adolescents Aged 2–17 Years, by Race/Ethnicity, Medical Expenditure Panel Survey, 2001 and 2016^a^

Variable	Prevalence of Use, 2001, % (95% CI)	Prevalence of Use, 2016, % (95% CI)	Absolute Disparity in 2001^b^, Percentage Point (95% CI)	*P *Value	Absolute Disparity in 2016^b^, Percentage Point (95% CI)	*P* Value	Change in Disparity from 2001 to 2016^c^, Percentage Point (95% CI)	*P* Value
**Sample size, n**	8,242	8,520	—	—	—	—	—	—
**All**	47.8 (46.0−49.6)	54.8 (52.8−56.8)	—	—	—	—	—	—
**Race/ethnicity**								
Non-Hispanic white	56.8 (54.4−59.2)	59.8 (56.8−62.8)	Reference		Reference		Reference	
Non-Hispanic black	31.4 (28.0−34.9)	44.1 (40.4−47.9)	25.4 (20.9−29.9)	<.001	15.7 (10.5−20.9)	<.001	−9.7	.006
Hispanic	33.3 (30.3−36.4)	50.7 (47.7−53.8)	23.6 (19.9−27.2)	<.001	9.1 (4.0−14.1)	<.001	−14.4	<.001
Asian	38.1 (30.8−45.9)	55.2 (48.8−61.3)	18.8 (11.8−25.6)	<.001	4.7 (3.1−12.4)	.238	−14.1	.008

Abbreviations: —, not applicable.

a Healthy People 2020 target for prevalence in dental care use is 49%.

b Absolute disparity is percentage-point difference in prevalence of use between non-Hispanic white and other groups: 56.8%−31.4% = 25.4 (non-Hispanic black in 2001).

c Change in disparity is percentage-point difference in absolute disparity at most recent data point subtracted from absolute disparity at baseline data point: for example, 15.7−25.4 = −9.7 (for non-Hispanic black children and adolescents in 2001–2006).

### Bivariate analysis

**Population characteristics by race/ethnicity**. Non-Hispanic white and Asian children had similar high proportions of parents or guardians with more than a 12th-grade education, high annual incomes, and private health insurance. In all racial/ethnic groups, the proportions of children with private health insurance and dental insurance decreased over time while the proportions with public health insurance increased. Non-Hispanic black children and Hispanic children had the greatest increase in public health insurance.

**Prevalence of dental care use by population characteristics**. From the first data cycle (2001–2005) to the third data cycle (2011–2016), a slight but significant increase occurred in the proportion of children using dental care (Table 2). At each data cycle, non-Hispanic white children had the highest use, whereas Asian children’s use fell between that of non-Hispanic white children and the other groups. We saw significant increases in percentage of use from 2001 to 2016 for non-Hispanic black children, from 36.8 % (95% CI, 35.0%–38.7%) to 44.4% (95% CI, 42.6%–46.2%); Hispanic children, from 36.2% (95% CI, 34.6%−37.8%) to 47.9% (95% CI, 46.3%−49.5%); and Asian children, from 43.2% (95% CI, 39.4%−47.0%) to 52.0 (95% CI, 48.2%−55.7%). Non-Hispanic white children had no significant increase. In addition, significant increases in use were found among children with parents or guardians at the lowest education level (*P* < .001), among low-income children (*P* < .001), and among publicly insured children (*P* < .001).

**Table 2:** Prevalence of Dental Care Use by Population Characteristics (Weighted Proportions), US Children Aged 2–17 Years (N = 132,763), by Data Cycle (2001−2005, 2006−2010, and 2011−2016), Medical Expenditure Panel Survey, 2001 and 2016

Variable	2001−2005 Use, % (95% CI)	2006−2010 Use, % (95% CI)	2011−2016 Use, % (95%CI)	*P* Value for Change From 2001–2005 to 2011–2016
**Eligible sample size, n**	43,760	39,744	49,259	—
**Proportion with ≥1 visit**	50.1 (49.0–51.2)	50.8 (49.7–51.9)	53.5 (52.3–54.8)	<.001
**Race/ethnicity^a^**				
Non-Hispanic white	58.3 (56.8–59.7)	56.9 (55.4–58.4)	58.8 (57.0–60.5)	.66
Non-Hispanic black	36.8 (35.0–38.7)	41.9 (40.0–43.7)	44.4 (42.6–46.2)	<.001
Hispanic	36.2 (34.6–37.8)	41.5 (39.9–43.1)	47.9 (46.3–49.5)	<.001
Asian	43.2 (39.4–47.0)	48.1 (43.0–53.6)	52.0 (48.2–55.7)	.001
**Age, y**				
2–4	26.5 (25.0–28.0)	29.7 (28.1–31.4)	34.4 (32.6–36.2)	<.001
5–11	56.3 (54.9–57.7)	56.5 (55.0–57.9)	58.0 (56.5–59.5)	.09
12–17	54.3 (52.9–55.7)	54.9 (53.4–56.4)	57.6 (55.9–59.2)	.002
**Sex**				
Male	49.0 (47.8–50.2)	49.6 (48.3–51.0)	52.7 (51.2–54.1)	<.001
Female	51.2 (49.9–52.6)	52.1 (50.8–53.4)	54.5 (53.1–55.8)	<.001
**Parent or guardian’s education**				
<12th grade	30.4 (28.6–32.1)	36.8 (34.7–38.9)	42.7 (40.6–44.8)	<.001
12th grade	42.5 (40.9–44.0)	42.2 (40.4–44.0)	44.9 (42.9–46.8)	.05
>12th grade	59.8 (58.5–61.2)	57.8 (56.4–59.1)	58.3 (56.9–59.7)	.14
**Annual household income^b^**				
Poor/low income	36.0 (34.9–37.1)	39.7 (38.4–41.1)	44.9 (43.5–46.4)	<.001
Middle income	51.3 (49.5–53.1)	51.9 (50.4–53.5)	54.1 (52.3–55.9)	.03
High income	67.7 (66.0–69.3)	65.7 (63.6–67.7)	66.1 (64.2–68.0)	.14
**Health insuranc**e				
Any private	57.7 (56.5–59.0)	57.9 (56.5–59.3)	60.1 (58.5–61.6)	.03
Public only	37.4 (35.9–38.9)	41.6 (40.0–43.2)	46.3 (44.9–47.8)	<.001
Uninsured	26.2 (23.4–29.2)	29.1 (25.9–32.5)	28.7 (24.9–32.9)	.403
**Dental insurance**				
Any	58.2 (56.8–59.6)	59.1 (57.5–60.6)	61.0 (59.4–62.6)	.02
None	41.6 (40.3–42.9)	43.5 (42.2–44.8)	47.3 (46.0–48.6)	<.001

Abbreviation: —, not applicable.

a All tests for differences in dental care use between a racial/ethnic group and non-Hispanic white children and adolescents were significant based on a 2-sided *t* test at *P* <.05 level of significance.

b Defined as percentage of federal poverty level: low income, <200%; middle income, 200%−399%; high income, ≥400% (1).

### Multivariate analysis

The sample for our multivariate analysis was 128,141 children. The decrease from 132,763 in sample size used in the bivariate analysis was due to missing data on parent or guardian’s education. We cross-tabulated private health insurance and private dental insurance by using a subset of 2005 data for 8,755 children. Among the 3,266 of those children with dental insurance, 98% had private health insurance, and among 4,122 children with private health insurance, 77.7% had dental insurance. Because of the high correlation between private dental insurance and private health insurance (R = 0.72, Pearson correlation coefficient), dental insurance was excluded as a covariate in the multivariate analysis. This decision was further supported by an assessment of multicollinearity that showed large variance proportions (greater than 0.5) for health (0.82) and dental (0.72) insurance.

We ran the same model for low-, medium-, and high-income levels, because household income modified the effect of race/ethnicity on dental care use. After we controlled for covariates, non-Hispanic black children had lower use than non-Hispanic white children at all income levels (low income, aPR = 0.84 [95% CI, 0.81−0.88]; middle income, aPR = 0.79 [95% CI, 0.75−0.83]; and high income, aPR = 0.80 [95% CI, 0.75−0.85]) (Table 3). Low-income Hispanic children (aPR = 0.98; 95% CI, 0.94−1.02) and low-income Asian children (aPR = 0.92; 95% CI, 0.83−1.02) showed no difference in use relative to non-Hispanic white children. The only group that showed a significant increase in use over time was low-income children of all race/ethnicities. Using the first data cycle as a reference (2001−2005) showed that use increased significantly by 8.0% in the second data cycle (aPR = 1.08; 95% CI, 1.03−1.13) and increased significantly by 18% in the third data cycle (aPR = 1.18; 95% CI, 1.12−1.24).

**Table 3:** Adjusted Prevalence Ratio Estimates for Factors Associated With Dental Care Use Among US Children and Adolescents Aged 2–17 Years (N = 128,141)^a^, by Income Level^b^, Using Pooled Years of Data (2001−2005, 2006−2010, 2011−2016), Medical Expenditure Panel Survey, 2001 and 2016

Variable	Low Income, aPR (95% CI)	Middle Income, aPR (95% CI)	High Income, aPR (95% CI)
**No. of observations used in analysis**	72,893	33,414	21,834
**Age, y**			
2–4	1 [Reference]	1 [Reference]	1 [Reference]
5–11	1.70 (1.61–1.79)^c^	2.12 (2.01–2.24)^c^	1.97 (1.86–2.07)^c^
12–17	1.59 (1.51–1.68)^c^	2.06 (1.95–2.18)^c^	1.98 (1.87–2.09)^c^
**Sex**			
Male	1 [Reference]	1 [Reference]	1 [Reference]
Female	1.06 (1.03–1.08)^c^	1.04 (1.02–1.07)^c^	1.02 (1.00–1.05)
**Race/ethnicity**			
Non-Hispanic white	1 [Reference]	1 [Reference]	1 [Reference]
Non–Hispanic black	0.84 (0.81–0.88)^c^	0.79 (0.75–0.83)^c^	0.80 (0.75–0.85)^c^
Hispanic	0.98 (0.94–1.02)	0.87 (0.84–0.91)^c^	0.85 (0.81–0.89)^c^
Asian	0.92 (0.83–1.02)	0.79 (0.73–0.86)^c^	0.82 (0.78–0.88)^c^
**Parent or guardian’s education**			
<12th grade	1 [Reference]	1 [Reference]	1 [Reference]
12th grade	1.10 (1.05–1.14)^c^	1.05 (0.97–1.14)	1.02 (0.87–1.19)
>12th grade	1.23 (1.17–1.29)^c^	1.30 (1.21–1.40)^c^	1.25 (1.07–1.45)^c^
**Health insurance**			
Uninsured	1 [Reference]	1 [Reference]	1 [Reference]
Any private	2.23 (1.99–2.50)^c^	1.68 (1.53–1.84)^c^	1.29 (1.17–1.42)^c^
Public only	2.20 (1.97–2.47)^c^	1.53 (1.37–1.70)^c^	1.15 (1.01–1.31)^c^
**Data cycle**			
2001–2005	1 [Reference]	1 [Reference]	1 [Reference]
2006–2010	1.08 (1.03–1.13)^c^	1.00 (0.97–1.05)	0.97 (0.94–1.01)
2011–2016	1.18 (1.12–1.24)^c^	1.02 (0.98–1.07)	0.98 (0.94–1.01)

Abbreviations: aPR, adjusted prevalence ratio.

a Total study participants was 132,763; however, only 128,141 had data on parents’ education.

b Income defined as percentage of federal poverty level: low income, <200%; middle income, 200%−399%; high income, ≥400% (1).

c Indicates significant at *P* < .05.

The association between dental care use and public health insurance compared with no insurance varied by income (low income, aPR = 2.20 [95% CI, 1.97−2.47]; middle income, aPR = 1.53 [95% CI, 1.37−1.70]; and high income, aPR = 1.15 [95% CI, 1.01−1.31]). We found similar results with children with private health insurance compared with those who were uninsured (low income, aPR = 2.23 [95% CI, 1.99−2.50]; middle income, aPR = 1.68 [95% CI, 1.53−1.84]; and high income, aPR = 1.29 [95% CI, 1.17−1.42]).

## Discussion

The Healthy People 2020 methodology provides a straightforward way of monitoring changes in disparities. By using the 2001 baseline prevalence, future changes in disparities can be easily assessed as subsequent years of crude use estimates from MEPS data are released. Our data on disparities and multivariate analyses showed the same result, that non-Hispanic black children made the least progress in reducing disparities relative to non-Hispanic white children. This result supported our hypothesis that either non-Hispanic black children or Hispanic children would have lower dental care use and the lowest changes in disparities relative to non-Hispanic white children. Hispanic children showed more progress than non-Hispanic black children in disparities reduction relative to non-Hispanic white children. However, the overall reduction in disparities was small and occurred only in the low-income group. Notably, disparities persisted for non-Hispanic black children at all income levels and for Hispanic and Asian children at middle- and high-income levels. Compared with non-Hispanic white children, Asian children had persistently lower use at middle- and high-income levels despite being similar to non-Hispanic white children in characteristics that positively influence dental care use, including high levels of parent or guardian’s education, household income, and private insurance. Our finding of lower use among Asian children was confirmed in a 2003−2004 national survey, which cited contributing factors as parents’ reports of problems obtaining specialty care and reports that the dentist did not know how to provide care (6). Several previous studies showed that expansion of public insurance for low-income children helped reduce disparities (5,7,11,21). Our findings showed that health insurance was 1 factor that positively influenced use, and its influence was greater for low-income children than for middle- and high-income children. Our study was not designed to directly assess the role of insurance in reduction of racial/ethnic disparities. However, the disparity reduction observed in our study may have been associated with the increase in the proportion of low-income children covered by public insurance over the study’s duration. Coverage with private insurance did not significantly increase from 2001 through 2016. Prevalence ratios were similar for the association of public and private health insurance with increased use compared with no health insurance, which highlights the importance of both types of insurance.

Another of our findings confirmed by previous studies was that disparities persisted and could not be explained by the variables available in the MEPS data set, including traditional sociodemographic factors or insurance (5,8,10). Persistent disparities could be explained by racial differences in oral health literacy, language, acculturation, and perception of need (2,22,23). Oral heath literacy is the ability to understand basic oral health information and the health care system to make appropriate health decisions. Low oral health literacy has been associated with a greater level of racial/ethnic disparities in oral health (22). Policy issues, such as dentist participation in public insurance programs, low reimbursement rates for public programs, and cost sharing can also limit access to dental services among uninsured and publicly insured children (13,23).

Differences in results between our study and previous multiyear, controlled analyses of national data are found in the magnitude of disparity reduction in our study and in the magnitude of persistent disparities. A controlled study of the 1964‒2010 National Health Interview Survey (NHIS) data found that disparities in dental care use among non-Hispanic black relative to non-Hispanic white children were large and significant in 1996 but attenuated and became nonsignificant by 2010 (8). Our finding in MEPS data of persistent disparities between non-Hispanic black and non-Hispanic white children is not consistent with the findings in NHIS data. This inconsistency can be explained by differences in methodology in national surveys, resulting in different estimates of prevalence of dental care use (24). A 2001–2010 MEPS study using a decomposition regression analysis found dental care use relative to non-Hispanic white children increased to a greater extent for non-Hispanic black than for Hispanic children (10). In contrast, our study found that use relative to non-Hispanic white children increased to a greater extent for Hispanic than for non-Hispanic black children with low incomes. Variation in methodology in studies using the same data set can lead to different conclusions about how and why disparities have changed; therefore, it is important to study disparities with multiple methods (25). Another possible explanation for differences between our study and the 2001‒2010 MEPS study is that we used more recent data.

Our study has limitations. MEPS is a cross-sectional survey, so we were unable to infer causality. The results in our study are based on self-reported data, which could result in some social desirability or recall bias for dental care use, even though MEPS ascertains dental visits over a relatively short term (within 3 to 4 months) compared with other national surveys (within 1 year) (24). Our study had no measure of oral health status or perceived need for care, which are important predictors of use. Another limitation was our use of health insurance as a proxy for dental insurance. One-third of children with private health insurance did not have dental insurance and likely had lower use than those with dental insurance. This difference may have resulted in a bias to the null in the association between use and private health insurance.

Our study showed a modest increase in dental care use and narrowing of disparities for some low-income children. Use by low-income Hispanic and Asian children caught up with non-Hispanic white children. Nevertheless, progress has been minimal, because use in all low-income children remains well below that of middle- and high-income children. Disparities persisted for non-Hispanic black children at all income levels. Insurance appeared to be an important factor but did not eliminate disparities. It is important to continue to monitor progress in disparities reduction.

## References

[1] Berdahl TA , Friedman BS , McCormick MC , Simpson L . Annual report on health care for children and youth in the United States: trends in racial/ethnic, income, and insurance disparities over time, 2002–2009. Acad Pediatr 2013;13(3):191–203. 10.1016/j.acap.2013.02.003 23680339

[2] Institute of Medicine, National Research Council. Improving access to oral health care for vulnerable and underserved populations. Washington (DC): The National Academies Press; 2011.

[3] Griffin SO , Barker LK , Wei L , Li C-H , Albuquerque MS , Gooch BF ; Centers for Disease Control and Prevention (CDC). Use of dental care and effective preventive services in preventing tooth decay among U.S. children and adolescents — Medical Expenditure Panel Survey, United States, 2003–2009 and National Health and Nutrition Examination Survey, United States, 2005–2010. MMWR Suppl 2014;63(2):54–60. 25208259

[4] Gilbert GH , Shah GR , Shelton BJ , Heft MW , Bradford EH Jr , Chavers LS . Racial differences in predictors of dental care use. Health Serv Res 2002;37(6):1487–507. 10.1111/1475-6773.01217 12546283 PMC1464042

[5] Flores G , Lin H . Trends in racial/ethnic disparities in medical and oral health, access to care, and use of services in US children: has anything changed over the years? Int J Equity Health 2013;12(1):10. 10.1186/1475-9276-12-10 23339566 PMC3560223

[6] Flores G , Tomany-Korman SC . Racial and ethnic disparities in medical and dental health, access to care, and use of services in US children. Pediatrics 2008;121(2):e286–98. 10.1542/peds.2007-1243 18195000

[7] Shone LP , Dick AW , Klein JD , Zwanziger J , Szilagyi PG . Reduction in racial and ethnic disparities after enrollment in the State Children’s Health Insurance Program. Pediatrics 2005;115(6):e697–705. 10.1542/peds.2004-1726 15930198

[8] Isong IA , Soobader MJ , Fisher-Owens SA , Weintraub JA , Gansky SA , Platt LJ , Racial disparity trends in children’s dental visits: US National Health Interview Survey, 1964–2010. Pediatrics 2012;130(2):306–14. 10.1542/peds.2011-0838 22753556 PMC3408679

[9] Hughes DC , Duderstadt KG , Soobader MP , Newacheck PW . Disparities in children’s use of oral health services. Public Health Rep 2005;120(4):455–62. 10.1177/003335490512000413 16025726 PMC1497737

[10] Vujicic M , Nasseh K . A decade in dental care utilization among adults and children (2001–2010). Health Serv Res 2014;49(2):460–80. 10.1111/1475-6773.12130 24299620 PMC3976182

[11] Wall TP , Brown LJ . Public dental expenditures and dental visits among children in the U.S., 1996–2004. Public Health Rep 2008;123(5):636–45. 10.1177/003335490812300514 18828419 PMC2496937

[12] Ku L , Sharac J , Bruen B , Thomas M , Norris L . Increased use of dental services by children covered by Medicaid: 2000–2010. Medicare Medicaid Res Rev 2013;3(3):E1–12. 10.5600/mmrr.003.03.b01 24753975 PMC3983734

[13] Liao CC , Ganz ML , Jiang H , Chelmow T . The impact of the public insurance expansions on children’s use of preventive dental care. Matern Child Health J 2010;14(1):58–66. 10.1007/s10995-008-0432-3 19067137

[14] Decker SL . Medicaid payment levels to dentists and access to dental care among children and adolescents. JAMA 2011;306(2):187–93. 10.1001/jama.2011.956 21750296

[15] US Department of Health and Human Services. Healthy People 2020 topics and objectives. Oral health. https://www.healthypeople.gov/2020/leading-health-indicators/2020-LHI-Topics. Accessed April 17, 2020.

[16] US Department of Health and Human Services. Healthy People 2020 leading health indicators. Oral health. https://www.healthypeople.gov/2020/topics-objectives/topic/oral-health/objectives#5028. Accessed April 17, 2020.

[17] Keppel K , Pamuk E , Lynch J , Carter-Pokras O , Insun K , Mays V , Methodological issues in measuring health disparities. Vital Health Stat 2 2005;(141):1–16. 16032956 PMC3681823

[18] Tali M , Huang DT . Measuring progress toward target attainment and the elimination of health disparities in Healthy People 2020. Statistical Notes, no. 27. Hyattsville (MD): National Center for Health Statistics; 2016. 10.15620/cdc/164019 PMC12278040 40576483

[19] Pearcy JN , Keppel KG . Monitoring change in health disparity. J Public Health Manag Pract 2008;14(5):481–6. 10.1097/01.PHH.0000333884.30023.d6 18708893

[20] Agency for Healthcare Research and Quality. Medical Expenditure Panel Survey. MEPS HC sample sizes, 2019. http://meps.ahrq.gov/mepsweb/survey_comp/hc_sample_size.jsp. Accessed May 10, 2020.

[21] Shi L , Stevens GD . Disparities in access to care and satisfaction among U.S. children: the roles of race/ethnicity and poverty status. Public Health Rep 2005;120(4):431–41. 10.1177/003335490512000410 16025723 PMC1497738

[22] Horowitz AM , Kleinman DV . Oral health literacy: a pathway to reducing oral health disparities in Maryland. J Public Health Dent 2012;72(Suppl 1):S26–30. 10.1111/j.1752-7325.2012.00316.x 22433091

[23] Williams DR , Costa MV , Odunlami AO , Mohammed SA . Moving upstream: how interventions that address the social determinants of health can improve health and reduce disparities. J Public Health Manag Pract 2008;14(Suppl):S8–17. 18843244 10.1097/01.PHH.0000338382.36695.42 PMC3431152

[24] Macek MD , Manski RJ , Vargas CM , Moeller JF . Comparing oral health care utilization estimates in the United States across three nationally representative surveys. Health Serv Res 2002;37(2):499–521. 10.1111/1475-6773.034 12036005 PMC1430363

[25] Pourat N , Finocchio L . Racial and ethnic disparities in dental care for publicly insured children. Health Aff (Millwood) 2010;29(7):1356–63. 20606188 10.1377/hlthaff.2009.0089

